# Laser-assisted vascular welding: optimization of acute and post-hydration welding strength

**Published:** 2015-06-21

**Authors:** Dara R. Pabittei, Michal Heger, Marc Simonet, Sjoerd van Tuijl, Allard C. van der Wal, Ed van Bavel, Ron Balm, Bas A. J. M. de Mol

**Affiliations:** 1 Department of Cardio -thoracic Surgery, Academic Medical Center, University of Amsterdam, Amsterdam, the Netherlands; 2 Department of Surgery, Academic Medical Center, University of Amsterdam, Amsterdam, the Netherlands; 3 Department of Physiology, Faculty of Medicine, Hasanuddin University, Jl, Makassar, South Sulawesi, Indonesia; 4 Department of Experimental Surgery, Academic Medical Center, University of Amsterdam, Amsterdam, the Netherlands; 5 Department of Biomedical Engineering, Material Technology, Technical University Eindhoven, the Netherlands; 6 HemoLab, Den Dolech 2, 5612 AZ Eindhoven, the Netherlands; 7 Department of Pathology, Academic Medical Center, University of Amsterdam, Amsterdam, the Netherlands; 8 Department of Biomedical Engineering and Physics, Academic Medical Center, University of Amsterdam, the Netherlands

**Keywords:** non-invasive vascular surgery, cohesive and adhesive bonding, coaptation, albumin solder, polycaprolactone, poly(lactic-co-glycolic acid), biomaterial scaffold

## Abstract

**Background:**

Liquid solder laser-assisted vascular welding using biocompatible polymeric scaffolds (ssLAVW) is a novel technique for vascular anastomoses. Although ssLAVW has pronounced advantages over conventional suturing, drawbacks include low welding strength and extensive thermal damage.

**Aim:**

To determine optimal ssLAVW parameters for maximum welding strength and minimal thermal damage.

**Methods:**

Substudy 1 compared breaking strength (BS) of aortic strips welded with electrospun poly(ε-caprolactone) (PCL) or poly(lactic-co-glycolic acid) (PLGA) scaffold, 670-nm laser, 50-s single-spot continuous lasing (SSCL), and semi-solid solder (48% bovine serum albumin (BSA)/0.5% methylene blue (MB)/3% hydroxypropylmethylcellulose (HPMC)). Substudy 2 compared the semi-solid solder to 48% BSA/0.5% MB/0.38% genipin and 48% BSA/0.5% MB/3% HPMC/0.38% genipin solder. Substudy 3 compared SSCL to single-spot pulsed lasing (SSPL).

**Results:**

PCL-ssLAVW yielded an acute BS of 248.0 ± 54.0 N/cm^2^ and remained stable up to 7d of hydration. PLGA-ssLAVW obtained higher acute BS (408.6 ± 78.8 N/cm^2^) but revealed structural defects and a BS of 109.4 ± 42.6 N/cm^2^ after 14 d of hydration. The addition of HPMC and genipin improved the 14-d BS of PLGA-sLAVW (223.9 ± 19.1 N/cm^2^). Thermal damage was reduced with SSPL compared with SSCL.

**Conclusions:**

PCL-ssLAVW yielded lower but more stable welds than PLGA-ssLAVW. The addition of HPMC and genipin to the solder increased the post-hydration BS of PLGA-ssLAVW. SSPL regimen reduced thermal damage. PLGA-ssLAVW using 48% BSA/0.5% MB/3% HPMC/0.38% genipin solder and SSPL constitutes the most optimal welding modality.

**Relevance for patients:**

Surgical patients requiring vascular anastomoses may benefit from the advantages that ssLAVW potentially offers over conventional sutures (gold standard). These include no needle trauma and remnant suture materials in the patient, reduction of foreign body reaction, immediate liquid-tight sealing, and the possibility of a faster and easier procedure for minimally invasive and endoscopic anastomotic techniques.

## 1. Introduction

Laser-assisted vessel welding (LAVW) is an experimental technique that is being investigated as an alternative for suturing of end-to-end and end-to-side vascular anastomoses. The use of sutures, which currently comprises the gold standard approach, is associated with several drawbacks, including needle trauma, the introduction of exogenous materials (suture threads), induction of foreign body reactions, and leakage through the anastomosis [1-4]. Alternative mechanical closures, namely clips and tacks that are commonly used in minimally invasive and robotic cardiothoracic surgery, inflict more pronounced mechanical trauma and are associated with more extensive anastomosis-related bleeding. The drawbacks of mechanical closures can be circumvented by LAVW, which is non-invasive and does not mechanically damage the vascular wall [5], is therefore non-immunogenic [6], and provides an immediate liquid-tight seal [7, 8]. In addition to anastomosis of micro- and small-sized vessels, the water-tight sealing provided by LAVW has the potential to hemodynamically support large vessel anastomoses (i.e., as an adjunct sealant of gaps between sutures) and to aid in intravascular sealing of endovascular prosthesis [1].

Modern approaches to LAVW are based on the thermal coagulation of a chromophore-containing protein solder that is placed over the coapted vascular segments, referred to as solder-mediated LAVW (sLAVW) [9-13]. The coaptation is irradiated with a laser that emits a wavelength preferentially absorbed by the solder-embedded chromophore. The absorbed radiant energy is converted to heat, which diffuses throughout the solder and upper portion of vascular tissue, inflicting thermal denaturation of the superior vascular wall and solder. Thermal denaturation is accompanied by chemical bonding between unfolded proteins upon cooling [2]. The extent to which these interprotein bonds are formed dictates the strength of the weld [2, 10].

The ultimate goal of LAVW is to achieve welding strengths well above malignant hypertension pressure (> 250 mmHg), measured by the bursting pressure (mmHg) or breaking strength (N/cm^2^). Welding strength depends on the adhesive strength (the strength of the solder-tissue bond) and the cohesive strength (the strength of the tissue-tissue bond and inter-solder bonding). sLAVW produces welding strengths that exceed > 250 mmHg [14].

To further enhance welding strength, LAVW can be performed with a scaffold in combination with a protein solder, referred to scaffold-enhanced sLAVW (ssLAVW). In this approach, a semi-porous scaffold composed of biocompatible polymeric material is drenched in chromophore-containing solder, placed over the coaptation, and irradiated. The scaffold provides an intertwining fiber network that, after laser irradiation, solidifies/fortifies the thermally coagulated solder. ssLAVW produces greater welding strengths than sLAVW due to a greatly enhanced cohesive strength [15-23]. To date, poly(lactic-co-glycolic acid) (PLGA) and poly(ε-caprolactone) (PCL) have been the two most commonly used (co) polymers in ssLAVW [15-23]. PCL is typically favored over PLGA insofar as PCL use is not associated with the formation of acidic byproducts following LAVW [19-23]. However, the low melting point of PCL (60 °C, i.e., the approximate temperature at which collagen and albumin start to denature) causes the scaffold to shrink, inducing a diametrical mismatch between the upper and lower part of the vessel that, in the clinical setting, may lead to aneurysm formation [20, 24]. Thermal affliction of the PCL scaffold is also associated with a reduction in E-modulus, yield strength, and ultimate strength [20].

PLGA has a higher melting point than PCL. Depending on the glycolic:lactic acid ratio in the copolymer, the melting point of PLGA varies from 173 °C (ratio of 5:95) to 201 °C (ratio of 90:10) [25]. Because such temperatures are never reached during ssLAVW, PLGA is expected to remain intact and may therefore produce higher welding strengths than PCL. The first aim of the study was therefore to determine which scaffold material (PCL vs. PLGA) is most optimal in terms of welding strength, mechanical and physical properties, and post-hydration weld stability, using the previously established ssLAVW protocol [19-21].

A persistent challenge in (ss)LAVW has been to improve adhesive bonding while minimizing thermal damage to the vascular wall. It was shown that the addition of a protein cross-linker, genipin, to solid albumin solder yields a significant increase in welding strength due to cross-linking of albumin to tissue collagen [26, 27]. Accordingly, the second aim of the study was to examine the fortifying effects of genipin in PCL and PLGA ssLAVW and to determine the ultrastructural quality and post-hydration stability of the welds.

In previous work [21] it was demonstrated that single spot ssLAVW produces stronger welds than scanning ssLAVW, but that the thermal damage extended to 2/3 of the medial layer as a result of the long pulse duration. The extensive thermal damage may limit future application of ssLAVW. Using the optimal ssLAVW modality (based on the results related to the second aim), the third aim of the study was therefore to define an optimal lasing regimen that produced maximum welding strength at minimal thermal damage. Single spot lasing was performed in continuous irradiation and pulsed irradiation mode and correlated to temperature profiles at the solder-tissue (s-t) interface. Subsequently, thermal damage, welding strength, and post-hydration weld stability were analyzed. The cooling interval between sequential pulses was expected to translate to reduced thermal damage to the vascular wall without impeding adhesive strength.

## 2. Materials and Methods

The concentrations listed throughout the manuscript refer to final concentrations. Supplemental figures are indicated with a prefix ‘S.’

### 2.1. Tissue preparation

Fresh porcine aortas (n = 40) were harvested at the slaugh-terhouse. Aorta strips were stored in PBS at 4 °C and were used within 3 d. Perivascular tissue was trimmed and the aortas were cut along the longitudinal axis. Subsequently, 30 × 5-mm longitudinal strips (n = 530) were punched from the unfolded slabs of vascular tissue.

### 2.2. Preparation of PCL and PLGA scaffolds

All scaffolds were electrospun in a climate-controlled electrospinning cabinet (IME Technology, Eindhoven, the Netherlands) equipped with a 14-G capillary and a distance of 15 cm between capillary and the target drum (25 × 120 mm). PCL scaffolds were prepared as described previously [19-21]. Briefly, PCL (M_w_ = 120,000, CAPA 6800, Perstorp UK, Cheshire, UK) was dissolved in chloroform ata 17% (w/w) concentration. The polymer solution was electrospun for 25 min at 23 °C and 50% relative humidity with a flow rate of 60 μL/ min and 15 kV to produce a mesh consisting of fibers with a diameter range of 12-14 μm.

PLGA (intrinsic viscosity = 1.8, Purasorb PLG 82.18, PuracBiochem, Gorinchem, the Netherlands) scaffolds with different fiber diameter were produced as follows. A 15% (w/w) PLGA in chloroform solution was electrospun at 23 °C and 50% relative humidity at 20 kV using a flow rate of 50 μL/min for 26-μm fibers and 35 μL/min for 19-µm fibers. Meshes with 10-µm diameter fibers were electrospun from a 13% (w/w) chloroform solution at 23 °C and 50% relative humidity with a flow rate of 35 μL/min and 20 kV. The same 13% (w/w) solution was electrospun for 60 min at 23 °C and 40% relative humidity with 16 kV and 20 μL/min to produce scaffolds with a 14-µm fiber diameter.

Fiber diameter was confirmed by scanning electron microscopy (SEM, Quanta 600F ESEM-FEG, FEI Company, Hilsboro, OR) using analytical software (Xt Microscope Control, FEI Company). For mechanical testing and ssLAVW, scaffolds were punched out of the mesh in 20 × 5-mm and 5 × 5-mm arrays, respectively. Scaffold thickness was measured with a digital micrometer between two glass slides.

### 2.3. Solder preparation

Semi-solid albumin solder consisted of 48% (w/v) bovine serum albumin (BSA, Fraction V, Roche, Penzberg, Germany), 0.5% (w/v) methylene blue (MB, Sigma-Aldrich, St. Louis, MO), and 3% hydroxypropylmethylcellulose (HPMC, Sigma-Aldrich) in MilliQ water [21, 28]. The semi-solid solder is designated as BSA-HPMC solder from here onward. Of note, MB has the advantage of turning white upon heating (i.e., undergo a transition to its leuco-form), which switches off MB-mediated heat production and hence deters extensive over-heating during irradiation. The leucoform transition property of MB and its beneficial implications on peri-irradiation thermodynamics were the reasons for choosing MB over other available chromophores.

For preparation of the BSA-genipin and BSA-HPMC-genipin solder, genipin was dissolved in ice-cold MilliQ (1% (w/v) final concentration) for 24 h under continuous stirring and at 4 °C until a homogenous suspension was obtained. Next, the genipin solution was placed in a water bath at 37 °C for 30 min to maximally dissolve the genipin [26, 29]. The BSA- genipin solder was prepared by mixing 48% (w/v) BSA and 0.5% (w/v) MB with the genipin solution (0.38% (v/v) final genipin concentration). The BSA-HPMC-genipin solder was prepared by mixing 48% (w/v) BSA, 0.5% (w/v) MB, and 3% (w/v) HPMC with the genipin solution (0.38% (v/v) final genipin concentration). All solders were stored at 4 °C in the dark for up to 1 week.

### 2.4. ssLAVW procedure

Aorta strips (n = 530) were pinned to a slab of silicone rubber (Sylgard 184, Dow Corning, Midland, MI) and placed in a petri dish. The aorta strips were cut in half along the longitudinal axis and realigned, after which 50 μL of solder and scaffold was applied over the adventitial surface of the incised strip.

A 670-nm diode laser (model HPD7401, High Power Devices, North Brunswick, NJ) was used in continuous wave mode for ssLAVW with a low-power red HeNe laser aiming beam. For mechanical testing and structural analysis only (section 2.5.1.1 and 2.5.1.2, respectively), irradiation of the scaffold was performed by dual pass scanning [20] because the minimal scaffold dimensions required for the stress and strain experiments exceeded that of the laser spot. The incident laser power was 0.7 W with a spot size of 12.6 mm^2^, accounting for an irradiance of 5.8 W/cm^2^. The scan speed was dictated by MB transiting into its leuco-form, i.e., irradiation of the subsequent scaffold volume was performed only after the scaffold area had turned white. This lasing regimen was standardly applied twice per scaffold.

Single spot continuous lasing (SSCL) was used for ssLAVW in all other experiments. The lasing modality was performed by positioning the laser probe at a 3.0-cm distance perpendicular to the scaffold surface to generate a spot diameter of 1.3 cm. The laser power was set to 1.6 W and irradiation was performed for 50 s, yielding an irradiance of 1.2 W/cm^2^ and a cumulative radiant exposure of 60 J/cm^2^. To prevent thermal damage to neighboring tissue, a black metal panel containing an 8 × 8-mm rectangular window was placed on the aorta strip before ssLAVW [21].

Modifications to the single spot lasing regimen to reduce thermal damage are explained in section 2.5.3.

### 2.5. Experimental design

#### 2.5.1. Determination of optimal scaffold material for ssLAVW

##### 2.5.1.1. Mechanical properties of PCL and PLGA scaffold before and after irradiation

Fifty-six scaffolds (n = 7/treatment/scaffold) were prepared for the determination of the stress–strain relation. Native electrospun PCL and PLGA scaffolds were tested without any modification as control. Solder-soaked scaffolds were prepared by soaking scaffolds in 1 mL of BSA-HPMC solder dispersed over a small area in a petri dish. Scaffolds were gently dabbed to enhance solder penetration. To investigate the effect of irradiation on the solder-soaked scaffold, PCL and PLGA scaffolds underwent a similar soaking procedure, after which they were subjected to dual pass irradiation at 5.8 W/cm^2^ (section 2.4). Lastly, to study the effect of heating exclusively on the scaffolds’ fibers (without albumin solder), PCL and PLGA scaffolds were submersed for ~2 min in 0.5% (w/v) MB in MilliQ prior to dual pass laser irradiation.

Mechanical tests were performed on scaffolds using a tensiometer (n = 7/group). The force of the tensiometer was zeroed prior to mounting the scaffolds into the metal clamps. The grip-to-grip separation was set at 5 mm and the test was performed at a test speed of 5 mm/min. Stress-strain curves during mechanical testing were made to compare mechanical behavior between PCL and PLGA scaffolds, while E-modulus, yield strength, and yield stress were calculated in MatLab (MatLab R2011b, MathWorks, Natick, MA) to determine ssLAVW- induced alterations of the mechanical properties.

##### 2.5.1.2. Thermal profiles and structural properties of PCL and PLGA scaffolds before and after irradiation

The melting points of PCL and PLGA scaffolds were measured using a differential scanning calorimetry (DSC) (DSC 823e, Mettler-Toledo International, Columbus, OH). DSC scans were performed at 10 °C/min from 0 to 120 °C for PCL scaffolds and from 0-200 °C for PLGA scaffolds. The 2^nd^ heating was performed to determine the melting point.

Scaffolds (n = 3/treatment/scaffold) were prepared and irradiated as described in section 2.5.1.1, after which they were examined by SEM analysis (section 2.7).

##### 2.5.1.3. Optimization of PLGA scaffold properties for ssLAVW

To define the optimal scaffold properties of PLGA scaffolds, PLGA was spun at different electrospinning settings to produce scaffolds with a fiber diameter of 10, 14, 19, or 26 μm and a thickness between 120-200 μm (section 2.2). Scaffolds were cut into sections of 5 × 5 mm. ssLAVW was performed with BSA-HPMC solder and single spot lasing (n = 7-10/ group). Breaking strength (BS) analysis was conducted as described in section 2.6. The combination of fiber diameter and scaffold thickness at which the highest BS was obtained was considered optimal for ssLAVW. These optimized scaffolds were then used in subsequent studies.

##### 2.5.1.4. Breaking strength analysis PCL and PLGA ssLAVW

After determination of the optimal scaffold properties of PCL [19] and PLGA (section 2.5.1.3), the scaffolds were subjected to BS analysis (section 2.6) following ssLAVW with BSA-HPMC solder (section 2.4). Twenty ssLAVWed specimens underwent BS analysis directly after ssLAVW as the 0-d control group (n = 10/scaffold). Sixty ssLAVWed aorta specimens were submersed in sterile PBS and incubated at 37 °C for 1, 7, or 14 d (n = 10/hydration period/scaffold). SEM analysis (section 2.7) was performed at the end of the hydration period (n = 3/hydration period/scaffold) to determine the structural features of the weld at the s-t interface.

#### 2.5.2. Optimization of solder properties for PCL and PLGA ssLAVW

PCL and PLGA ssLAVW was performed by SSCL (section 2.4) with different solder compositions, namely BSA-HPMC, BSA-genipin, and BSA-HPMC-genipin. BS analysis (section 2.6, n = 10/solder) and SEM analysis (section 2.7, n = 3/hydration period) were performed at 0-, 1-, 7-, and 14 d of hydration (section 2.5.1.4). The solder-scaffold combination that yielded the highest and most stable welding strength was used in the experiments that followed.

In order to relate breaking strength and structural features of the weld to temperature evolution during ssLAVW, the temperature evolution during the ssLAVW procedure (n = 5/solder composition) was determined in a separate set of experiments. To avoid the melting of PCL fibers to the thermocouple (section 2.5.1.1), this part of the experiment was performed using PLGA scaffolds only. A type T thermocouple (Ø = 0.25 mm) was placed between the incised aortas, after which solder (BSA-HPMC, BSA-genipin, or BSA-HPMC-genipin) and scaffold were applied onto the incision. ssLAVW was performed as described in section 2.4 using a ~180-µm thick PLGA scaffold. The temperature at the s-t interface was recorded in real time before, during, and after ssLAVW with (PicoLog, Pico Technology, Cambridgeshire, UK*)*. Histology samples were prepared to investigate thermal damage to the vessel (section 2.8, n = 5/solder composition).

The denaturation/melting temperatures of BSA, MB, and all three solder compositions were measured by DSC. A sealed pan was used to reduce the background of water evaporation. DSC scans were performed at 10 °C/min from 30-95 °C. A difference measurement was obtained by completing two sequential scans for a single sample. The first scan was performed to denature solders, whereas the second scan was performed to obtain a background thermal profile for the remaining denatured sample. The thermal difference between the first and the second scan was defined as irreversible denaturation of the solder [30].

#### 2.5.3. Continuous versus pulsed single spot ssLAVW

In order to reduce thermal damage, single spot ssLAVW was performed with BSA-HPMC-genipin solder, PLGA scaffold, and with either 50-s SSCL (section 2.5.2) or with single spot pulsed lasing (SSPL). The following lasing regimens were explored (n = 23/lasing regimen), where the laser pulse duration is given in seconds and the intermittent cooling time (in s) is provided between vertical lines: 25-|5|-15; 25-|10|-15; 25-|15|-15; 25-|10|-10-|10|-10; and 25-|10|-15-|10|-10. BS tests (section 2.6) were performed directly after ssLAVW on n = 10 samples/lasing regimen. To assess the adhesive bond, n = 3 samples/lasing regimen were prepared and analyzed under SEM (section 2.7). Histology samples (section 2.8, n = 5/lasing regimen) were prepared to qualify the extent of thermal damage to the vessel wall. Temperature evolution during each lasing regimen was recorded in 5 samples/lasing regimen in separate sets of experiments (section 2.5.2). Hydration experiments were performed on the lasing regimen groups that produced comparable BS as the control group (25-|10|-15 and 25-|10|-15-|10|-10) and the group that exhibited the least thermal damage (25-|15|-15). Hydration experiments were performed as described in section 2.5.1.4 (n = 10/hydration period/group). BS analysis (section 2.6) and SEM analysis (section 2.7, n = 3/hydration period/lasing regimen) were also performed at the end of the hydration period.

### 2.6. Breaking strength analysis

BS tests were performed after every 5 consecutive ssLAVW procedures, with the exception of the hydration experiments, where BS testing was performed directly on all hydrated samples. The ssLAVWed aorta strips were kept moist in PBS-soaked gauzes between the ssLAVW procedure and BS testing. The strips were mounted in the metal clamps of a tensiometer (Zwick Z101, Ulm, Germany). The distance between the clamps was 10 mm. BS analysis was performed at a test speed of 10 mm/min. BS was defined as the force (N) required to completely tear two halves of the strip, divided by the cross-sectional area (in cm^2^) of the scaffold and solder. The cross-sectional area of the solder was measured right before the breaking strength (BS) measurement was performed (after welding and after hydration period).

### 2.7. Scanning electron microscopy

The native, solder-soaked, irradiated solder-soaked, MB-soaked, and irradiated MB-soaked PCL and PLGA scaffolds were viewed under SEM without prior fixation and coating. To eliminate residual water, scaffolds were kept under vacuum overnight after preparation and before SEM. The scaffold strip was cut in a 5 × 5-mm array and mounted to a metal stump before placement inside the SEM. Scaffolds were viewed under low vacuum at an accelerating voltage of 10 kV.

SEM analysis was also performed to investigate the structure of adhesive bonding at the s-t interface in fixed specimen cross-sections. Immediately following ssLAVW or at the end of the hydration period, the samples were fixed in 1.5% glutaraldehyde, cut in half along the longitudinal axis to expose the s-t cross-section, and desiccated by CO_2_ critical point drying. The dried sample was mounted on a metal stump and placed inside the SEM. Adhesive bonding was analyzed in a high vacuum system at an accelerating voltage of 1 kV.

### 2.8. Thermal damage analysis

Histological samples were prepared from ssLAVWed aortas in the 2^nd^ and 3^rd^ substudies described in section 2.5.2 and 2.5.3, respectively. Immediately after ssLAVW, vessel segments were fixed in 4% buffered formalin, dehydrated in ethanol, cleared in methyl benzoate and benzene, and impregnated with paraffin wax. Of each sample, 6-μm sections were stained with Masson’s trichrome (MT) for light microscopy and with picrosirius red (PR) for polarization microscopy. Images were acquired with an Olympus microscope (model BX51, Osaka, Japan) equipped with a polarizer and analyzer and an Olympus DP70 camera. Olympus imaging software was used for image acquisition and processing.

### 2.9. Statistical analysis

Means, standard deviations, standard error of the means, Mann-Whitney U tests, Kruskal-Wallis tests, two-tailed homoscedastic Student’s t tests, and ANOVA tests were performed in GraphPad Prism (GraphPad Software, La Jolla, CA). When differences were found in the multiple comparison tests, a Bonferron (ANOVA) or Dunn’s (Kruskal-Wallis) post-hoc test was performed. When comparing various treatment regimens to a control group (i.e., native scaffold, 0-d hydration group, or 50-s single spot continuous lasing) a Dunnet’s post hoc multiple comparison test was employed. The choice for parametric or nonparametric testing was based on the D’Agostino Pearson omnibus test of normality, which was performed on each data set. The number of symbols designates the strength of the p-value, i.e., (*), (**), and (***) designate a p-value of ≤ 0.05, ≤ 0.01, and ≤ 0.001, respectively, throughout the text.

## 3. Results

### 3.1. Determination of optimal scaffold material for ssLAVW

#### 3.1.1. Mechanical properties of PCL and PLGA scaffold before and after irradiation

**Figure 1.**
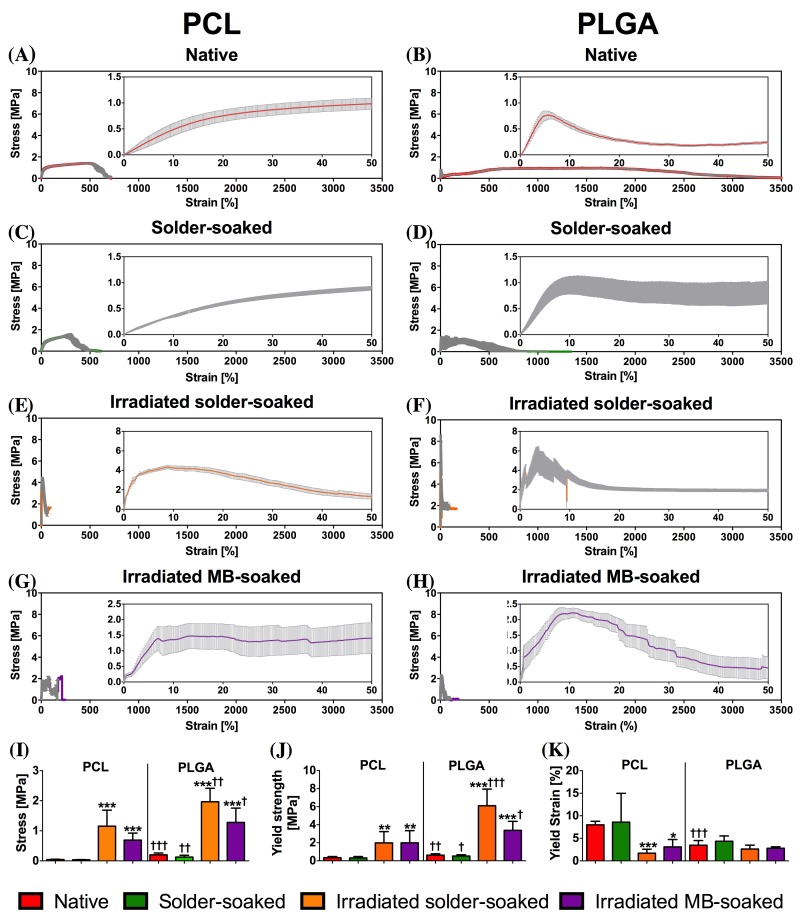
Mean ± standard error of the mean (SEM) stress-to-strain curves of native (A, B), solder-soaked (C, D), irradiated solder-soaked (E, F), and irradiated MB-soaked (G, H) PCL and PLGA scaffolds (n = 7 per experiment). The inset represents a magnification of the 0-50% strain range. The bar charts represent the associated mechanical properties: E-modulus (I), yield strength (J), and yield strain (K). An (*) designates the level of significance versus native scaffold, while (†) designates the level of significance between the similarly treated PCL and PLGA scaffolds.

Figure 1 depicts the stress-to-strain curves of native, solder-soaked, irradiated solder-soaked, and irradiated MB-soaked PCL and PLGA scaffolds. The embedded graphs present mechanical properties of both scaffold types under different conditions. Based on the E-modulus, yield stress, and yield strain, native PLGA scaffolds were considerably stiffer and stronger than PCL scaffolds (†††). Moreover, PCL scaffolds gradually increased in strength before breaking at approximately 1,000% of strain (Figure 1A), whereas PLGA scaffolds rapidly increased in strength, underwent plastic deformation, and dropped in strength when the fiber packing was disrupted. Following this drop, the PLGA fibers realigned in the direction of the traction and rebuilt strength until completely breaking at 3,000% of the original strain. Presoaking in solder did not affect the mechanical properties of either type of scaffold. Dual-pass irradiation significantly increased the strength and stiffness of solder-soaked PCL and PLGA scaffolds (Figure 1E and F). However, post-irradiation PLGA scaffolds were stronger and stiffer than PCL scaffolds^1^, as evidenced by the higher E-modulus (††, Figure 1I) and yield strength (†††, Figure 1J) of the former.

The effect of heating on scaffold properties was investigated in the MB-soaked scaffold group. In the absence of a solder, the irradiated PLGA scaffold remained stiffer and stronger than PCL. The E-modulus of MB-soaked PLGA and PCL scaffolds was 1.3 ± 0.5 MPa and 0.7 ± 0.2 MPa (†, Figure 1I), respectively, while the yield strength was 3.4 ± 1.0 MPa and 2.0 ± 1.3 MPa (†, Figure 1J), respectively.

#### 3.1.2. Thermal profiles and structural properties of PCL and PLGA scaffolds before and after irradiation

**Figure 2:**
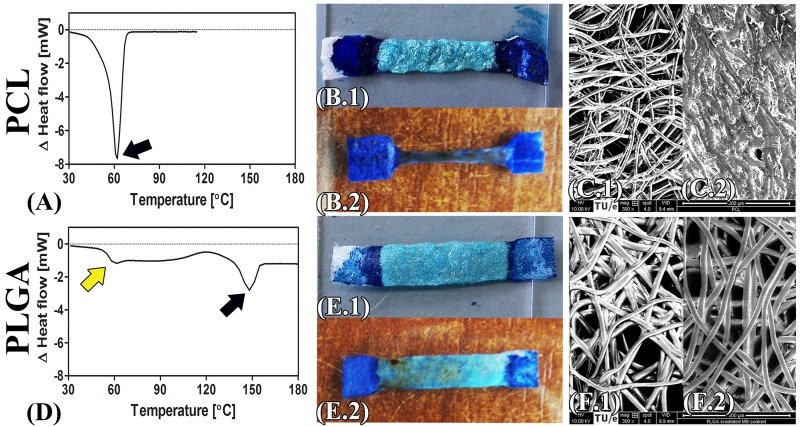
DSC graphs of PCL (A) and PLGA (D) scaffold. Black arrows indicate the melting points, whereas the yellow arrow designates the glass transition temperature. Macroscopic images of the irradiated solder-soaked PCL (B.1) vs. PLGA (E.1) scaffolds and irradiated MB-soaked PCL (B.2) vs. PLGA (E.2) scaffolds. Respective SEM images are included of MB-soaked PCL (C.1) vs. PLGA (F.1) scaffolds and irradiated MB-soaked PCL (C.2) vs. PLGA scaffolds (F.2).

To determine the effect of laser-induced heating on the scaffold material, PCL and PLGA scaffolds were soaked in solder or MB, irradiated, and investigated macroscopically and by SEM. Macroscopic images of irradiated solder-soaked and MB-soaked PCL vs. PLGA scaffolds are presented in Figure 2, demonstrating that the MB-containing solder exerts a deterrent effect on PCL scaffold shrinkage (Figure 2B.1). In the absence of solder, the low melting point of PCL (T_m_ = 62 °C, Figure 2A) was associated with scaffold shrinkage to 70% of the original width (Figure 2B.2). SEM analysis of the MB-soaked scaffold revealed melted and coagulated PCL fibers (Figure 2C.2). PLGA scaffolds, on the other hand, have a higher melting point (T_m_ = 148 °C, Figure 2D) that caused these scaffolds to retain their structure following irradiation. Macroscopically, no notable differences were observed between the irradiated solder-soaked and irradiated MB-soaked PLGA scaffolds (Figure 2E.1 and 2E.2). Intact and preserved PLGA fibers were observed after irradiation by SEM (Figure 2F.2).

The complete set of SEM images of native, solder-soaked, and irradiated solder-soaked PCL and PLGA scaffolds is presented in Figure S1.

#### 3.1.3. Optimization of PLGA scaffold properties for ssLAVW

**Figure 3.**
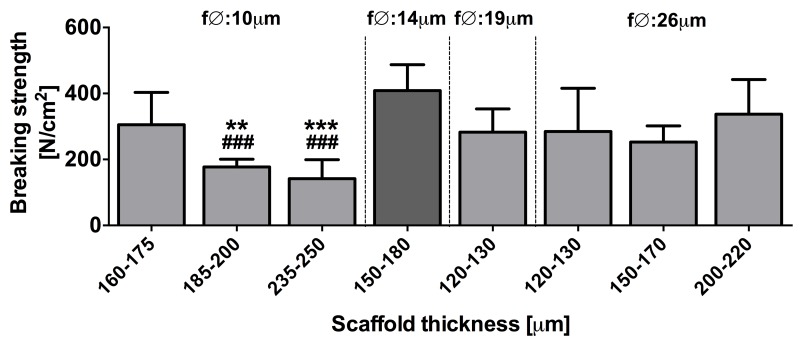
Mean ± SD acute BS of PLGA ssLAVW for different combinations of fiber diameter and scaffold thickness (n = 8-12/group). ssLAVW was performed with BSA-HPMC solder and single spot irradiation. The group that produced the highest BS is demarcated in dark gray. (*) designates the statistical significance in the 10-µm fiber diameter group versus the 160-175-μm scaffold thickness group, whereas (#) indicates the level of significance versus the group with the highest BS (14-μm fiber diameter).

Next, the ssLAVW modality was optimized for PLGA scaffolds using single spot irradiation, as was performed for PCL previously [19]. Figure 3 presents acute BS after PLGA ssLAVW using different combinations of fiber diameter and scaffold thickness. At a 10-μm fiber diameter, scaffolds with a thickness of 160-175 μm produced the highest welding strength (305.3 ± 98.1 N/cm^2^), whereby the BS decreased with increasing scaffold thickness. For scaffolds with a fiber diameter of 26 μm, an increased scaffold thickness had no impact on welding strength. The 19-μm fiber diameter group yielded comparable welding strength as the 26-μm fiber diameter group. The combination of 14-μm fiber diameter and a scaffold thickness of 150-180 μm produced the highest acute BS, and was therefore used in the subsequent substudies.

#### 3.1.4. Breaking strength analysis PCL and PLGA ssLAVW

**Figure 4.**
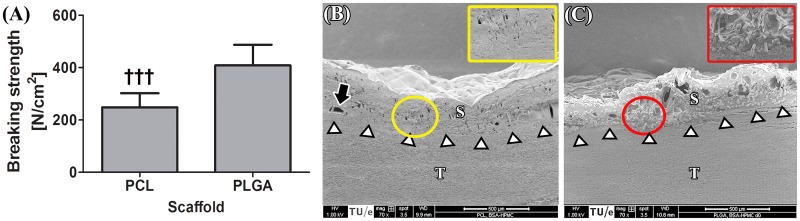
Acute BS of PCL vs. PLGA ssLAVW (n = 10/group) (A) and SEM images of cross-sectional areas of PCL (B) and PLGA (C) ssLAVWed aorta strips. The inset represents a 150 × magnification of the encircled area. White arrowheads are pointing to the solder (S)-tissue (T) interface, black arrow is pointing to a focal solder-tissue gap, yellow circle indicates the melted PCL fibers, and red circle demarcates the intact PLGA fibers.

For an experimental ssLAVW modality to succeed in the clinical setting, it must produce the highest possible welding strength. In the next set of experiments, the welding strengths obtained with PCL and PLGA ssLAVW were compared so as to identify the most suitable scaffold material. PLGA ssLAVW produced higher acute welding strength than PCL ssLAVW (Figure 4A), accounting for 408.6 ± 78.8 N/cm^2^ and 248.0 ± 54.0 N/cm^2^, respectively. SEM imaging revealed seamless adhesive bonds in both PCL and PLGA ssLAVWed aortas (white arrows, Figure 4B and 4C). As reported in section 3.1.2, PCL fibers had melted and coalesced with the coagulated solder (Figure 4B, inset), whereas PLGA fibers were intact and interspersed throughout the coagulated solder (Figure 4C, inset).

**Figure 5.**
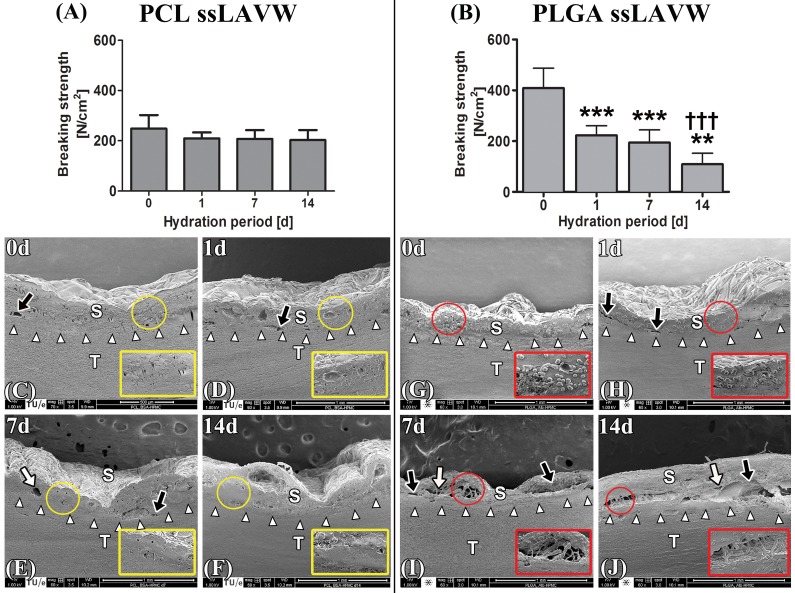
BS of PCL (A) and PLGA (B) ssLAVWed aorta strips plotted as a function of hydration period (n = 10/time point). Aortic strips were welded using BSA-HPMC solder and single spot irradiation. BS tests and SEM were performed after 1-, 7-, and 14 d of hydration. BS tests and SEM images taken directly after ssLAVW were used as the 0-d control specimens. The SEM images represent cross-sectional areas of PCL (C-F) and PLGA (G-J) ssLAVWed aortas on hydration days 0, 1, 7, and 14, respectively. White arrowheads point to the s-t interface, black arrows indicate separation gaps between the coagulated solder (S) and tissue (T), and white arrows designate the cavitations in the solder/scaffold coagulum. Red circles highlight intact PLGA fibers. An (*) designates the level of significance vs. 0 d of hydration and a (†) reflects the level of intergroup significance at the same time point.

In vivo, welds may be susceptible to deterioration due to e.g., hydrolysis. The stability of the welds was therefore tested under quasi-physiological conditions by hydration in sterile PBS at 37 ºC. PCL ssLAVW remained stable up to 7 d of hydration, a reduction in BS was observed at the end of the 14-d hydration period (*, Figure 5A). SEM analysis confirmed good adhesive bonding in the welds, with only small separation gaps occurring after 14 d of hydration (Figure 5C-F). PCL fibers appeared to have coalesced with the coagulated solder (Figure 5C-F, insets).

A marked decrease in post-hydration BS was observed in PLGA ssLAVWed aortas (Figure 5B). The mean ± SD BS decreased by 47%, 52%, and 73% relative to t = 0 d following 1-, 7-, and 14 d of hydration, respectively. SEM analysis on PLGA ssLAVWed aortas showed considerable separation gaps at the s-t interface (Figure 5H-J, black arrows) and cavitations in the solder/scaffold coagulum after 14 d of hydration (Figure 5J, white arrows). Intact PLGA fibers were observed between the coagulated solder until the end of the hydration period (Figure 5G-J, insets).

### 3.2. Optimization of solder properties for PCL and PLGA ssLAVW

In an effort to further optimize ssLAVW welding strengths, PCL and PLGA ssLAVW were performed with solder containing HMPC (semi-solid solder), genipin (protein cross-linker), or both. HMPC and genipin were shown to improve welding strengths in intestinal welding [26, 29].

**Figure 6.**
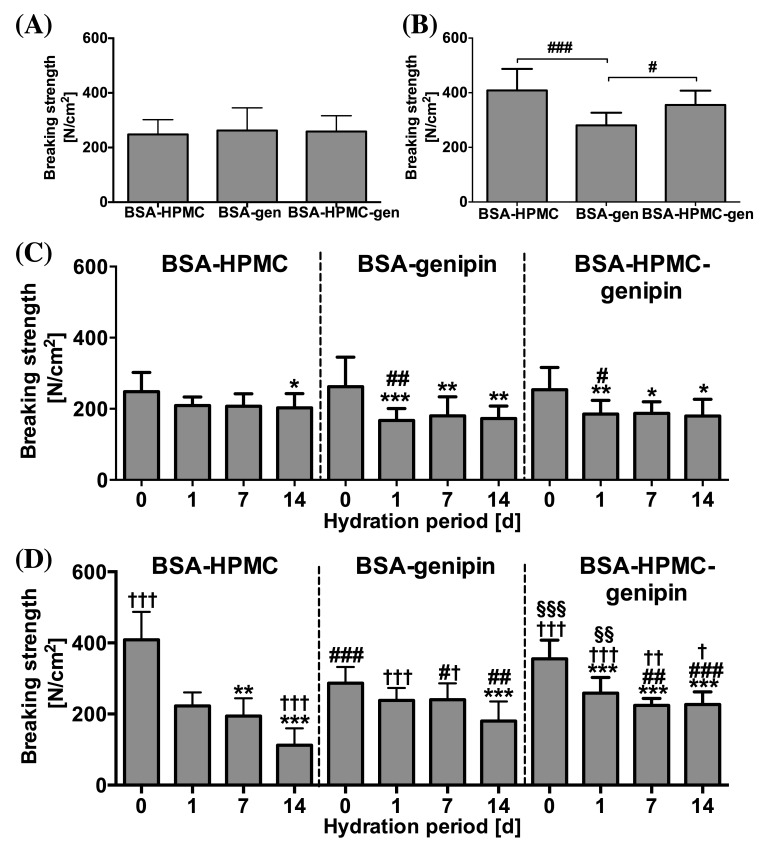
Mean ± SD acute BS of PCL(A) and PLGA(B) ssLAVW plotted as a function of solder composition and post-hydration BS of PCL (C) and PLGA (D) ssLAVW using various solder compositions plotted as a function of hydration time. Each bar shows the mean ± SD of 10-12 repairs. In B, (#) designates the level of significance between different solder compositions. In C and D, (*) indicates the level of significance vs. 0 d of hydration in the same group, (#) designates the intragroup level of significance between the genipin-enriched solder groups and the BSA-HPMC subgroup of the same scaffold type and hydration time, (†) defines the level of significance of PCL and PLGA subgroups of the same solder compositions and hydration time, and (§) represents the level of significance between PCL + BSA-HPMC vs. PLGA + BSA-HPMC-genipin at the same hydration time.

Irradiation of MB- and genipin-containing solder caused the solder to turn dark purple rather than white (leuco-form of MB), as was the case with MB-containing solder. Figure 6A and B present the acute BS of PCL and PLGA ssLAVW using different solder compositions. In PCL ssLAVWed aortas, the addition of genipin did not produce significant changes in acute BS (p = 0.83, Figure 6A). The addition of genipin to either BSA and BSA-HPMC solders is associated with a decrease in of BS after 1 d of hydration in PCL ssLAVW groups. (Figure 6C).

In the PLGA ssLAVW group, genipin-enriched BSA solder produced weaker welds than the BSA-HPMC solder (280.7 ± 46.0 N/cm^2^ vs. 408.6 ± 78.8 N/cm^2^, respectively, Figure 6B), but the welds produced with genipin solder remained stable for up to 7 d of hydration. Although a 30% decrease in BS was found after 14 d of hydration (*** versus 7-d hydration, Figure 6D), the welds with the genipin solder were associated with a greater 14-d post-hydration BS than the respective welds in the BSA-HPMC group (##). The addition of genipin to BSA-HPMC solder produced comparable acute BS as the BSA-HPMC solder, namely 355.1 ± 52.6 N/cm^2^ versus 408.6 ± 78.8 N/cm^2^ (ns), respectively. The welding strengths during 14 d of hydration were higher in the PLGA + BSA-HPMC-genipin group compared to the PLGA + BSA-HPMC group, comprising 258.7 ± 43.9 N/cm^2^, 209.0 ± 24.4 N/cm^2^, and 223.9 ± 19.1 N/cm^2^ for 1-, 7-, and 14 d of hydration, respectively. These welding strength were similar to those achieved in the PCL scaffold group using BSA-HMPC solder, which produced welding strengths of 209.0 ± 24.4 N/cm^2^ (§§), 207.0 ± 35.1 N/cm^2^ (ns), and 202.9 ± 39.6 N/cm^2^ (ns), respectively.

**Figure 7.**
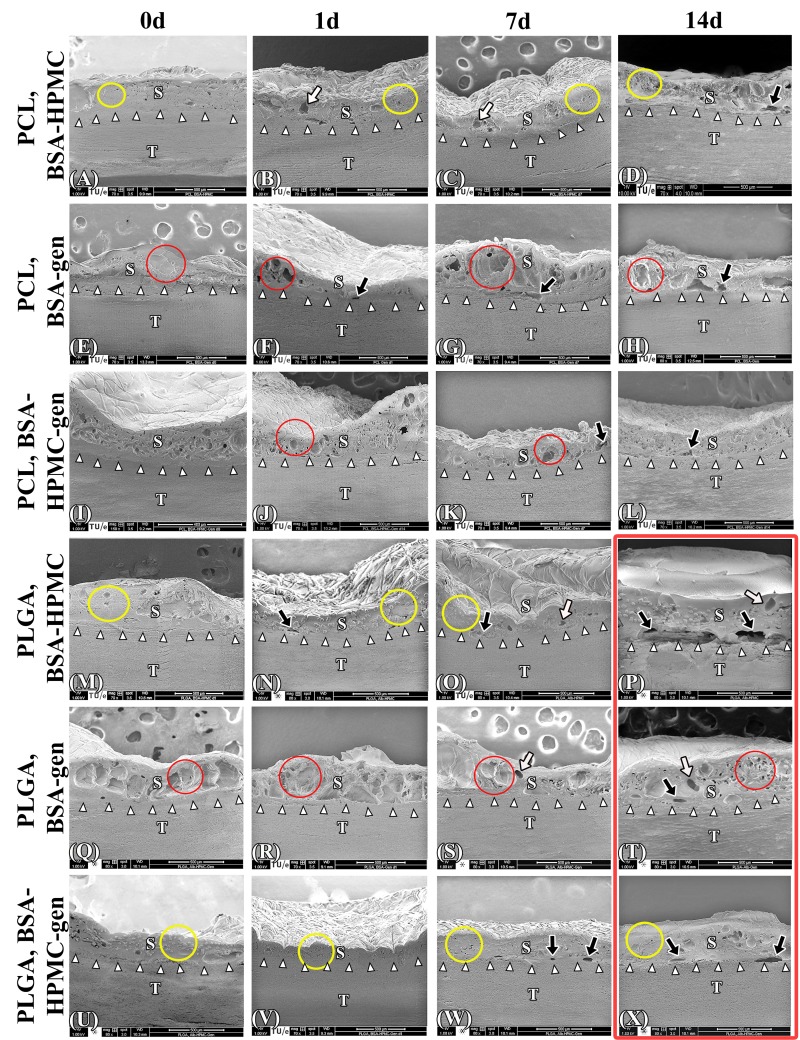
Acute and post-hydration SEM images of the cross-sectional areas of PCL and PLGA ssLAVWed aortas welded with different solders. White arrowheads, black arrows, and white arrows are pointing to the solder (S)-tissue (T) interface, the gaps at the s-t interface, and the cavitations at the solder/scaffold coagulum, respectively. Yellow circles highlight the solid parts of the solder/scaffold coagulum and red circles indicate the reticulated parts of the solder/scaffold coagulum. Gen = genipin.

In contrast to a solidified solder/scaffold coagulum observed in PCL+ BSA-HPMC ssLAVWed specimens (Figure.7A-D, yellow encircled), SEM imaging of genipin-enriched BSA PCL ssLAVWed specimens (Figure 7E-H) revealed a reticulated solder/scaffold coagulum (red encircled) containing multiple focal zones of separation (black arrows). A reticulated solder/scaffold coagulum was also observed in the PCL + BSA-HPMC-genipin group (Figure 7I-L, yellow encircled), but the coagulum contained a minimum amount of separations (Figure 7K and L, black arrows).

The improvement in post-hydration BS in the PLGA ssLAVW groups with genipin was reflected in the ultrastructural features of the weld. Compared to the PLGA + BSA-HPMC group (Figure 7M-P), the welds made with PLGA and BSA-genipin solder showed seamless adhesive bonding up to 7 d of hydration (Figure 7Q-S, white arrowheads) and a minimum amount of clefts at the s-t interface after 14 d of hydration (Figure 7T, black arrows). However, the solder/scaffold coagulum appeared reticulated in the solder part and contained numerous focal clefts (Figure 7Q-T, red encircled and white arrows, respectively). The combination of HPMC and genipin in the PLGA group produced a more solid solder/scaffold coagulum (Figure 7U-X, yellow encircled). Only few focal zones of separation were observed at the s-t interface in the PLGA + BSA-HPMC-genipin solder group after 7 d and 14 d of hydration (Figure 7W-X, black arrows).

The s-t temperatures generated during PLGA ssLAVW with solders of different composition and the corresponding photomicrographs of the PR-stained specimens are provided in Figure S2. PLGA ssLAVW with BSA-genipin solder induced steeper temperature increases in the first 15 s of irradiation than with BSA-HPMC solder, namely 5.2 °C/s and 4.4 °C/s, respectively (**). Upon irradiation, a dark purple color change occurred at the center of the laser spot and expanded laterally. After this color change, from 15-25 s the temperature increased gradually at a rate of 0.91 °C/s, 0.89 °C/s, and 0.7 °C/s for BSA-genipin, BSA-HPMC, and BSA-HPMC-genipin solders, respectively. In the last 25 s, the temperature increased at a much slower rate (0.2 °C/s) in all solders and the color change had extended over the entire scaffold area. At the end of the 50-s SSCL, the highest temperature at the s-t interface was achieved in the BSA-genipin solder group, i.e., 93.3 ± 4.2 °C (* vs. 79.9 ± 4.9 °C for BSA-HPMC and * vs. 84.7 ± 4.9 °C for BSA-HPMC-genipin). The aortas welded with BSA- genipin solder exhibited the most extensive thermal damage (Figure S2C.1 and C.2), which corresponds to the temperature profile (Figure S2C). Thermal profiles of BSA, MB, BSA-HPMC, BSA-genipin, and BSA-HPMC-genipin are presented in Figure S3.

### 3.3. Continuous versus pulsed single spot ssLAVW

**Figure 8.**
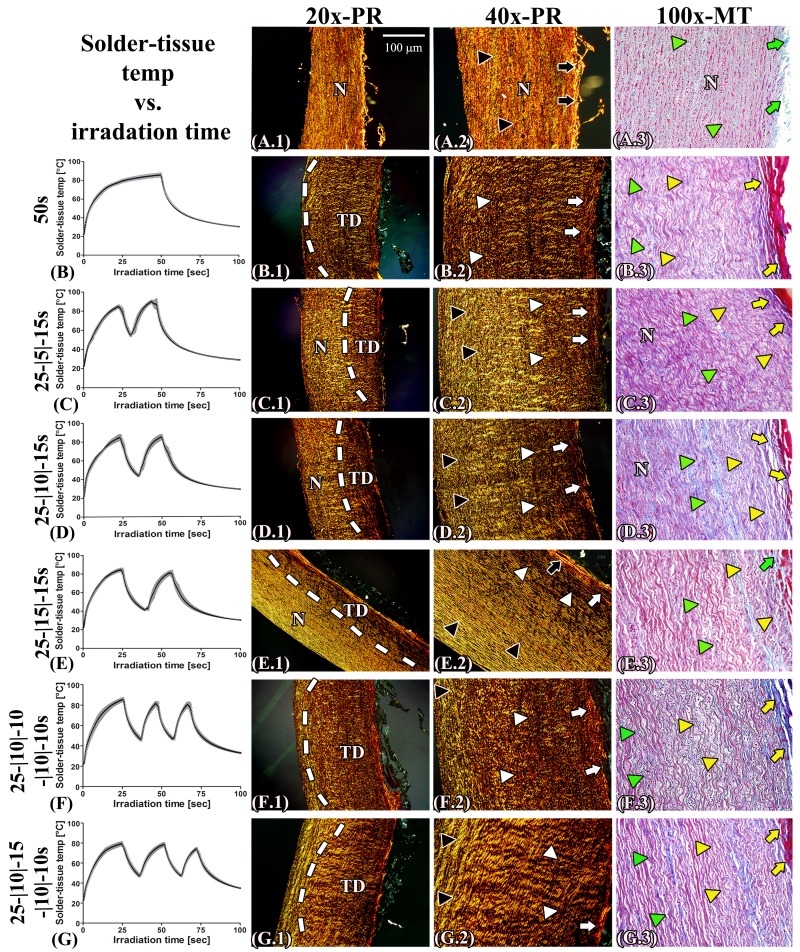
The mean ± SEM s-t temperature of PLGA ssLAVWed aortas during continuous (B) and single spot pulsed regimens (C, D, E, F, and G) plotted as a function of irradiation time with the corresponding photomicrographs depicting the inflicted thermal damage. Native (non-irradiated) aorta (A.1-3) was used as control. Low magnification images of PR-stained samples (B.1, C.1, D.1, E.1, F.1, and G.1) viewed under polarized light illustrates the extent of thermal damage. The white dashed line demarcates the border between the thermally damaged area (TD) and unaffected tissue (N). At higher magnification, the PR-stained control aorta (A.2) exhibited intact (birefringent) collagen in the adventitia (black arrow) and media (black arrowhead). In irradiated samples, the denatured adventitial collagen appeared homogenized and compacted (white arrows), while the loss of yellow-stained type I collagen was characteristic of thermally damaged media (white arrowheads). MT-stained control aorta (A.3) consisted of adventitia with loosely arranged collagen (green arrow) and intact smooth muscle cells in the media (green arrowhead). Thermally damaged aortas (B.3, C.3, F.3, D.3, E.3, F.3, and G.3) exhibited denatured red-stained solder that adhered to the adventitia, homogenized denatured collagen in the adventitia (yellow arrows), and loss of smooth muscle cells in the media (yellow arrowheads).In the upper left panel a scale bar was inserted, which represents 100 μm in the 20 × images, 50 μm in the 40 × images, and 20 μm in the 100 × images.

**Figure 9.**
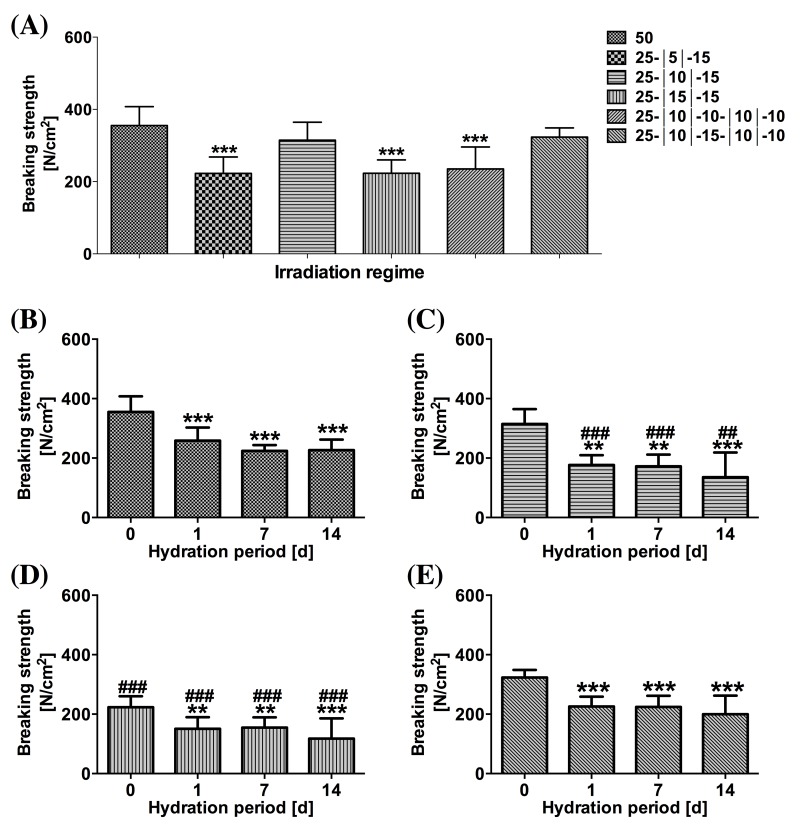
(A) Mean ± SD acute BS following PLGA ssLAVW plotted as a function of irradiation regimen. The post-hydration mean ± SD BS of 50 s (B), 25-|10|-15 s (C), 25-|15|-15 s (D), and 25-|10|-15-|10|-10 s (E) are also plotted as a function of hydration period. (*) designates the level of significance versus 0-d hydration and (#) indicates the level of significance of each time point versus the same time point of the control group.

**Figure 10.**
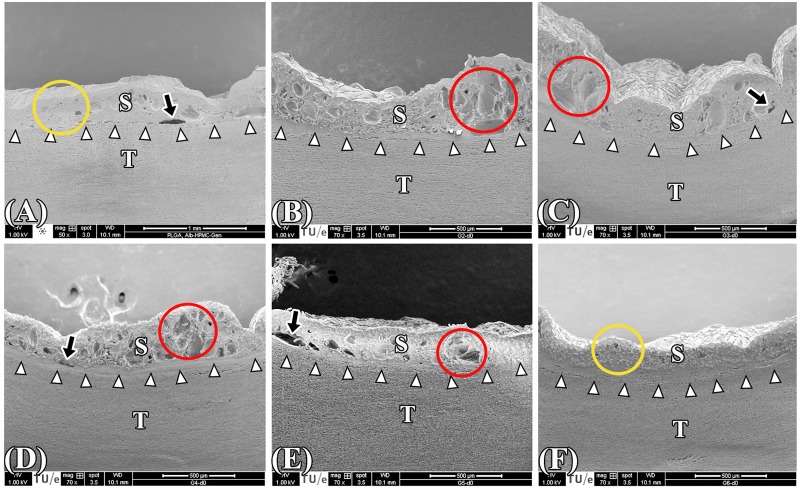
SEM images of the cross-sectional areas of PLGA ssLAVWed samples irradiated in 50-s continuous (A) and pulsed single spot regimens comprising 25-|5|-15 s (B), 25-|10|-15 s (C), 25-|15|-15 s (D), 25-|10|-10-|10|-10 s (E), and 25-|10|-15-|10|-10 s (F). White arrows point to the solder (S)–tissue (T) interface, black arrows indicate the gaps at the s-t interface, yellow circles designate the solid solder/scaffold coagulum, and red circles highlight the reticular segments in the solder/scaffold coagulum.

Figure 8 depicts temperature profiles at the s-t interface during SSCL and SSPL and the corresponding histology images. PR- and MT-stained native aortas were used as controls (Figure 8A.1-3). For the irradiated, PR-stained specimens, lower magnification images revealed the extent of thermal damage (Figure 8B.1, C.1, D.1, E.1, F.1, and G.1, white dashed line), whereas higher magnification images (Figure 8B.2, C.2, D.2, E.2, F.2, and G.2) showed homogenized and compacted thermally damaged adventitial collagen (white arrows) and loss of yellow-stained type I collagen in the thermally afflicted media (white arrowheads). Structural alterations in the aortic wall are demonstrated by the MT-stained samples (Figure 8B.3, C.3, D.3, E.3, F.3, and G.3), where compacted and homogenized collagens (yellow arrows) reflect thermally damaged adventitial tissue and the shrunken muscle cells (yellow arrowheads) indicate thermally damaged media. The respective mean ± SD acute BS and the SEM analysis are presented in Figure 9A and Figure 10, respectively.

Fifty-second SSCL raised the s-t temperature to 84.7 ± 5.2 °C (Figure 8B) and produced an acute BS of 355.1 ± 52.6 N/cm^2^ (Figure 9) that coincided with full thickness thermal damage (Figure 8B.1-3). A ~20 °C temperature gradient was created between the center and lateral sides of s-t interface during irradiation. Figure S4 depicts the temperature gradient and the corresponding thermal damage in both regions.

With respect to SSPL, the first pulse was terminated after 25s (in all single spot pulsing groups) on the basis of the observed color change. The mean ± SD s-t temperature after the first pulse was 80.7 ± 6.8 °C in all multiple pulse regimens. After a 5-, 10-, or 15-s cooling interval, the administration of a second pulsed raised the s-t temperature to 92.5 ± 4.9 °C, 85.7 ± 7.1 °C, and 82.9 ± 5.1 °C, respectively. The two-pulse regimens inflicted thermal damage up to 1/3 of the aortic wall (Figure 8C.1-3, D.1-3, and E.1-3). A third single spot pulse of 10 s or 15 s, following a 10-s cooling interval, induced thermal damage up to 2/3 of the aortic wall (Figure 8F.1-3 and G.1-3).

With respect to acute BS, only the 25-|10|-15 s and 25-|10|-15-|10|-10 s regimens obtained BSs that were comparable to the control group, namely 292.3 ± 59.3 N/cm^2^ and 323.4 ± 25.2 N/cm^2^, respectively (Figure 9A). However, SEM analysis revealed that solid solder/scaffold coagula were only achieved with the control and the 25-|10|-15-|10|-10 s regimens (Figure 10A and F, red circles). The s-t temperatures of the 25-|10|-15-|10|-10 s regimen were 79.7 ± 4.1 °C|45.8 ± 2.4 °C|78.9 ± 4.5 °C|46.6 ± 2.2 °C|74.6 ± 3.5 °C, respectively.

Hydration experiments were performed on the SSPL-subjected samples in which welding strengths were achieved that were comparable to the 50-s irradiation group (i.e., 25-|10|-15 s and 25-|10|-15-|10|-10 s) and in which the least thermal damage was found (i.e., 25-|15|-15 s). After 1-d of hydration, the BS in the control, 25-|10|-15 s, 25-|15|-15 s, and 25-|10|-15-|10|-10 s groups had decreased by 27%, 43%, 32%, and 21%, respectively (Figure 9B-E). Following 14d of hydration, welding strengths of the 25-|10|-15 s and 25-|15|-15 s groups had become considerably lower than the control group (Figure 9C vs.B), whereas the 25-|10|-15-|10|-10 s modality obtained comparable post-hydration BS relative to the control group (Figure 9E vs. B).

## 4. Discussion

The aim of this research was to optimize the previously described ssLAVW modality [21]. The study revealed the importance of intact fibers for the scaffolds’ mechanical strength and acute BS, but not for maintaining post-hydration BS. PLGA ssLAVW with BSA-HPMC solder produced higher acute BS than ssLAVW with PCL. However, the welds made with PLGA exhibited a reduction in strength with increasing hydration time, which in turn was ameliorated by the addition of genipin to the BSA-HPMC solder. As a result, ssLAVW with PLGA + BSA-HPMC-genipin yielded comparable post-hydration welding strength as PCL ssLAVW. Finally, it was shown that SSPL (versus SSCL) minimized collateral thermal damage and retained acute and post-hydration BS.

The thermo-mechanical properties of a biomaterial in part dictate the suitability of the biomaterial for a given application, particularly if the application is associated with the generation of high temperatures such as during (ss)LAVW. Due to the relatively low melting point of PCL (62 °C) versus PLGA (148 °C), it was expected that thermal denaturation of PCL fibers would compromise post-LAVW welding strength. Indeed, mechanical tests on PCL and PLGA scaffolds confirmed that PLGA was stronger than PCL after irradiation, both as free solder-impregnated scaffold and as a scaffold imposed on a vascular segment during ssLAVW. With respect to the latter, ssLAVW with PCL yielded an acute BS of 248.0 ± 54.0 N/cm^2^, which was significantly lower than the 408.6 ± 78.8 N/cm^2^ produced by PLGA ssLAVW. This corresponds to a breaking force of 1.4 ± 0.3 N for PCL ssLAVW^\^and 2.2 ± 0.4 N for PLGA ssLAVW. Earlier studies on ssLAVW with solvent casted particulate leached PLGA scaffolds reported breaking forces of 1.31 N [16], 1.5 ± 0.6 N [17], and 1.1 ± 0.2 N [18], which were all lower than the breaking force achieved in this study with comparable scaffold material. On the other hand, the breaking force of PCL ssLAVW achieved in this study was higher than an earlier report on PCL ssLAVW by Alfieri et al. (1.1 ± 0.2 N) [23] but lower than the 1.9 ± 0.4 N achieved in the study by Bregy et al. [22].

Despite the higher acute BS, aortas welded with PLGA + BSA-HPMC solder exhibited deterioration of welding strength under quasi-physiological conditions. Similar results have been reported by Sorg and Welch [16]. Although the exact mechanism responsible for the weld deterioration in PBS is currently elusive, the increase in the number of clefts in the scaffold/solder coagulum and the considerable separation at the s-t interface suggest water-induced retrogradation of cohesive and adhesive bonding. The fact that PCL is more hydrophobic [25] than PLGA may partly explain the relative absence of these phenomena in PCL-LAVWed coaptations subjected to 14-d hydration. The hydrophobic character of PCL apparently renders the scaffold material less amenable to water-induced loosening of cohesive and adhesive bonds. On the basis of these considerations and the post-irradiation yield strength of PCL and PLGA scaffolds, it can be concluded that (intact) PLGA fibers give rise to stronger cohesive bonding whereas (thermally denatured) PCL scaffolds yield more stable welds.

The improvement in post-hydration welding strength in PLGA ssLAVW and the corollary enhancement in adhesive bonding by the addition of genipin reflect the important role of genipin in promoting cross-linking between albumin and tissue collagen [26]. However, the reticulated solder/scaffold coagula observed in all BSA-genipin groups suggest excessive heat development in the solder/scaffold coagulum. The addition of HPMC to BSA-genipin solder produced lower s-t temperature and resulted in a more solid and stable solder/scaffold coagulum, yielding slightly higher acute and post-hydration BS. Ultimately, welds created with PLGA + BSA-HPMC-genipin produced post-hydration welding strengths that were comparable to those achieved with PCL ssLAVW. In a recently published study by our group [31], however, it was demonstrated that end-to-end anastomoses (porcine carotid arteries with an external diameter of 4.3-5.9 mm) that had been welded along the entire coapted circumference with BSA-HPMC-genipin PLGA scaffolds were more resilient in a 24-h pulsatile pressure test than welds made with BSA-HPMC PCL scaffolds. Despite the similar welding strengths achieved in this study with both types of hydration-subjected scaffolds, the 24-h pulsatile pressure test data [31] suggest that LAVA/R with BSA-HPMC-genipin PLGA scaffolds constitutes the most optimal combination. Nevertheless, ssLAVA experiments comparing the utility of BSA-HPMC-genipin PLGA scaffolds to BSA-HPMC PCL scaffolds must be performed in an in vivo proof-of-concept setting to arrive at a definitive conclusion, particularly since the weld degradation kinetics in vivo may differ from those in a quasi-physiological environment.

To determine the relationship between heat build-up at the s-t interface and welding strength as well as thermal damage, the temperature profiles during ssLAVW were determined for different welding regimens. The s-t temperature during a 50-s single spot laser pulse, namely 80-85 °C, was similar to values reported by Sorg and Welch [16]. This regimen, however, produced more extensive thermal damage than sLAVW, i.e., in the absence of scaffold [17]. Histology results showed considerable reduction of thermal damage using SSPL instead of SSCL. The cooling time allows tissue to thermally relax before the subsequent irradiation. In comparison to a 5- and 15-s cooling interval, the10-s cooling interval produced the most optimal results in terms of welding strength. Furthermore, despite the comparable acute BS achieved between the 25-|10|-15 s and control regimens, the two-SSPL regimen failed to retain post-hydration welding strength. Consequently, a third pulse was required to solidify the solder/scaffold coagulum and to increase the stability of the welds. The 25-|10|-15-|10|-10 s regimen reduced thermal damage to 2/3 of the aortic wall and produced comparable acute and post-hydration BS relative to SSCL (control). Moreover, the acute breaking forces obtained by 25-|10|-15-|10|-10 s and 25-|10|-15 s regimens, namely 1.7 ± 0.2 N and 1.5 ± 0.3 N (corresponding to a BS of 323.4 ± 25.2 N/cm^2^ and 292.3± 59.3 N/cm^2^, respectively), were considerably higher than the breaking force reported by Alfieri et al. for the 15-|15|-15 s regimen, namely 0.6 ± 0.04 N [23].

## 5. Conclusions and future outlook

In the final analysis, the previously described ssLAVW modality was optimized, but neither of the modalities proved to be superior over the other regarding post-hydration BS, the most important parameter for in vivo LAVW. Whereas PLGA ssLAVW yielded better acute cohesive bonding, PCL ssLAVW produced more stable welds. The quality of each modality should therefore be tested in ex vivo or in vivo models for further evaluation. Nevertheless, it is compelling to argue that a dual-layer scaffold composed of an inner PCL layer and an outer PLGA layer may provide more stable and stronger welds than ssLAVW with either biomaterial alone. Electrospinning allows the fabrication of a double-layered scaffold [32]. Also, genipin should be further explored as a weld-stabilizing agent, particularly when PLGA is used. An additional advantage of genipin is that it reduces an inflammatory response [33, 34], and may therefore be promising for clinical application especially when PLGA is used. Finally, the possibility of decreasing thermal damage while maintaining acute and post-hydration welding strength increases the potential of in vivo and clinical application of ssLAVW. However, application of end-to-end or end-to-side anastomosis requires modification of the laser beam to allow a 360° simultaneous and homogeneous irradiation. This type of lasing could be achieved by means of intraluminal irradiation with an isotropic beam [13] or external irradiation with several laser beams attached to a clip probe.

## Footnotes

1 Mechanical properties were calculated in MatLab in this study. E-modulus and yield strength of PCL scaffolds differed from the previously reported values [20], which were calculated with Zwick/Roell software using the autocalculation command.
